# An RT-PCR panel for rapid serotyping of dengue virus serotypes 1 to 4 in human serum and mosquito on a field-deployable PCR system

**DOI:** 10.1371/journal.pone.0214328

**Published:** 2019-03-25

**Authors:** Jih-Jin Tsai, Wei-Liang Liu, Ping-Chang Lin, Bo-Yi Huang, Ching-Yi Tsai, Pin-Hsing Chou, Fu-Chun Lee, Chia-Fong Ping, Pei-Yu Alison Lee, Li-Teh Liu, Chun-Hong Chen

**Affiliations:** 1 Center for Dengue Fever Control and Research, Kaohsiung Medical University, Kaohsiung, Taiwan; 2 School of Medicine, College of Medicine, Kaohsiung Medical University, Kaohsiung, Taiwan; 3 Tropical Medicine Center, Kaohsiung Medical University Hospital, Kaohsiung, Taiwan; 4 Division of Infectious Diseases, Department of Internal Medicine, Kaohsiung Medical University Hospital, Kaohsiung, Taiwan; 5 National Mosquito-Borne Diseases Control Research Center, National Health Research Institutes, Zhunan, Taiwan; 6 GeneReach Biotechnology, Taichung, Taiwan; 7 Department of Medical Laboratory Science and Biotechnology, College of Medical Technology, Chung-Hwa University of Medical Technology, Tainan City, Taiwan; 8 National Institute of Infectious Diseases and Vaccinology, National Health Research Institutes, Zhunan, Taiwan; National Yang-Ming University, TAIWAN

## Abstract

**Background:**

Dengue fever, a mosquito-borne disease, is caused by dengue virus (DENV) which includes four major serotypes (DENV-1, -2, -3, and -4). Some serotypes cause more severe diseases than the other; severe dengue is associated with secondary infections by a different serotype. Timely serotyping can provide early warning of dengue epidemics to improve management of patients and outbreaks. A mobile insulated isothermal PCR (iiPCR) system is available to allow molecular detection of pathogens near points of need.

**Methodology/Principle findings:**

In this study, side-by-side comparison with the CDC DENV-1-4 Real Time RT-PCR (qRT-PCR) was performed to evaluate the performance of four singleplex DENV-1–4 serotyping reverse transcription-iiPCR (RT-iiPCR) reagents for DENV subtyping on the mobile PCR system. The four RT-iiPCRs did not react with Zika virus and chikungunya virus; tests with serial dilutions of the four DENV serotypes made in human serum showed they had detection endpoints comparable to those of the reference method, indicating great analytical sensitivity and specificity. Clinical performance of the RT-iiPCR reagents was evaluated by testing 40 serum samples each (around 20 target serotype-positive and 20 DENV-negative); all four reagents had high agreement (97.5–100%) with the reference qRT-PCR. Moreover, testing of mosquitoes separately infected experimentally with each serotype showed that the four reagents detected specifically their target DENV serotypes in mosquito.

**Conclusions/Significance:**

With analytical and clinical performance comparable to the reference qRT-PCR assay, the four index RT-iiPCR reagents on the field-deployable PCR system can serve as a useful tool for DENV detection near points of needs.

## Introduction

Dengue virus (DENV), an enveloped virus with a single-stranded, positive sense RNA genome, belongs to the genus flavivirus and comprises mainly four serotypes (DENV-1, -2, -3, and -4). DENV infection causes a wide range of clinical signs in humans, from asymptomatic to acute febrile illness (dengue fever, DF), to severe hemorrhagic fever/dengue shock syndromes (DHF/DSS) [1]. Dengue disease is a major public health problem in developing tropical countries and has being continuously spreading to new geographical areas [2, 3]. Frequent international travel to dengue endemic or epidemic regions has contributed to the escalating numbers of imported dengue cases in temperate region. Outbreaks of the four DENV serotypes have been increasing reported in the tropics and sub-tropics mainly in Asia, South America, and the Caribbean; multiple virus serotypes have been found co-circulating in the hyperendemic regions in Southeast Asia and Pacific [2]. Taiwan, located in the tropical-subtropical region of the Northern Hemisphere, has seen many DENV outbreaks since the first half of 20th century. Since 2006, southern Taiwan has faced dengue outbreaks of different scales every year; relatively large outbreaks occurred in 2014 and 2015, with DENV-1 and -2 being the major serotype, respectively [4].

Diagnosis of DENV infection cannot rely solely on clinical signs and symptoms as the majority of the infected individuals are either asymptomatic or present with symptoms similar to those of other febrile-episode-inducing diseases [5]. DENV serotyping is important for disease management and public health surveillance. Several reports have indicated that DENV-2 and DENV-3 may cause more severe diseases and that DENV-4 is responsible for a milder illness than the other serotypes [6]. In addition, antibody-mediated enhancement (ADE) of DENV infection further complicates disease severity [7]. Chances for developing DHF-DSS is elevated when infection with one of the four serotypes is followed by a heterotypic serotype; the replacement of DENV-3 by DENV-1 in Sri Lanka in 2009, was associated with a wave of severe dengue epidemic in Sri Lanka [8–10].

DENV is transmitted to humans by mosquitoes (*Aedes aegypti* and *A*. *albopictus)*. *A*. *aegypti* can pick up DENV from people showing no symptoms or oligosymptom, resulting in silent transmission [11]. A positive association was established between DENV infection in humans and mosquitoes at very fine spatiotemporal scales in the natural setting; specifically, human cases were reported at about one week after positive *A*. *aegypti* in one study [12, 13].

Timely on-site detection and serotyping of DENV in human and mosquito can potentially alert front-line health professionals invasion of a new or long time absent serotype, allowing timely implementation of intervention strategies focuses in those areas to help mitigate disease outbreaks in human [2, 14]. Current methods to aid diagnosis of DENV infection include virus isolation (e.g. antigen detection immunofluorescence assay), nucleic acid amplification tests (NATs; e.g. reverse transcription-polymerase chain reaction [RT-PCR], real-time RT-PCR [qRT-PCR]), and serological assays (e.g. NS1 antigen detection, plaque reduction neutralization titers (PRNT), and enzyme linked immunosorbent assay [ELISA]) [15]. Although a number of NS1 rapid diagnostic tests are commercially available to detect NS1 antigen during the first few days of fever, they do not provide serotype information [16]. PRNT, antigen detection immunofluorescence assay are able to determine DENV serotypes [17], but they are both time-consuming, expensive, laborious, and feasible only in well-equipped laboratories. With relatively high specificity and sensitivity, NATs were recommended for the detection of DENV RNA by the World Health Organization [1]. Several multiplex qRT-PCR methods capable of serotyping have been reported [18–20]. However, performance of qRT-PCR tests requires skilled technicians and relatively expensive equipment that are not available to remote areas or developing countries; transportation of specimens is another major obstacle. In order to bring early serotyping of DENV to points of need (PON), a rapid, easy, mobile, NAT method of high sensitivity and specificity is needed.

Recently, the portable, simple and compact POCKIT Nucleic Acid Analyzer (POCKIT, GeneReach, Taichung, Taiwan) which can automatically detect and interpret PCR results within one hour became available in mobile PCR laboratory formats [21–23]. A lightweight hand-held model, POCKIT Micro Plus Nucleic Acid Analyzer (POCKIT Micro Plus), that works with a built-in rechargeable battery is also available. In this system, insulated isothermal PCR (iiPCR) is achieved consistently in a capillary tube (R-tube, GeneReach) in a simple, specially designed insulated heater and relies on fluorescent probe hydrolysis for signal detection [23, 24]. Various iiPCR/RT-iiPCR-based reagents, available commercially in a lyophilized format to facilitate long-term storage and easy shipping, have been validated to have analytical and clinical performance comparable to reference methods (real time PCR, nested PCR, virus isolation) for different important microbial pathogen hosts [25–31]. The POCKIT device has been bundled with easy field-deployable methods for nucleic acid extraction, with potential to serve as a flexible mobile PON tool for rapid DENV detection in human and mosquito.

A pan-DENV-specific RT-iiPCR assay was validated recently on the POCKIT system to have clinical performance equivalent to that of a laboratory qRT-PCR for the detection of DENV-1–4 serotypes in human plasma and serum [32, 33]. Four singleplex DENV serotyping RT-iiPCR reagents have become available recently for the identification of DENV-1, 2, 3, and 4 serotypes separately on the POCKIT system, allowing DENV serotyping near patients and soon after the mosquitoes are trapped even at low-resource settings. In this study, we evaluated the performance of the four DENV serotyping reagents on the POCKIT and POCKIT Micro Plus PCR devices for the detection of the respective target DENV serotypes in human serum and mosquito samples.

## Materials and methods

### Ethics statement

Serum samples were collected from clinically suspected dengue patients for routine diagnosis using RT-PCR methods [34] at the Tropical Medicine Center, Kaohsiung Medical University Hospital, Kaohsiung, Taiwan in 2012. The use of retrospective clinical specimens in this study was approved by the Kaohsiung Medical University Hospital Institutional Review Board (KMUHIRB-F(I)-20180009); waiver of informed consent was obtained. All collected samples were anonymized.

### Virus and mosquito samples

Tissue culture fluids containing DENV-1 (Hawaii strain), -2 (NGC strain), -3 (DN8700829A strain), or -4 (DN9000475A Strain) were collected after the viruses were propagated in the mosquito C6/36 cell line (*A*. *albopictus*). Zika virus strains (MR766, PRVABC59) were from the American Type Culture Collection, Manassas, VA, USA. Chikungunya virus (CK9500004) was from the Taiwan Center of Disease Control, Taipei, Taiwan.

The *A*. *aegypti* (UGAL) mosquito strain was used in this study and infected with DENV-1, DENV-2, DENV-3 or DENV-4 by micro-injection (nanoinjector) into the thoracic cavity. Adult female mosquitoes, aged 7–8 days, were cold anesthetized and inoculated using a microcapillary needle that had been pulled to a point with needle puller. The 4 serotype of dengue virus stocks were standardized to 2x10^6^ PFU/ml, and 0.2 μl was injected into each mosquito (approximately 400 PFU/mosquito). Infected mosquitoes were maintained in cages at 28 ± 1°C and 70% ± 5% relative humidity with 12h/12h light-dark cycle and fed with 10% sucrose solution. Three infected mosquitoes were collected every other day for the 7-day incubation period to determine the virus titers. The remaining mosquitoes were frozen and stored at −80°C until further use.

### Nucleic acid extraction

Nucleic acid extraction was performed by using the taco Preloaded DNA/RNA Extraction Kit (GeneReach) on a taco mini Automatic Nucleic Acid Extraction System (taco mini; GeneReach) according to the manufacturer’s instructions. Briefly, sample (200 μl) were added into the first well of the extraction plate and the automatic extraction steps were performed. All nucleic acids were subjected subsequently to the respective serotyping RT-iiPCR and the reference qRT-PCR systems in parallel. For mosquito samples, before taco mini extraction, each mosquito was homogenized in 250 μl PBS with a disposable grinder and centrifuged briefly. Subsequently, 200 μl of the upper aqueous sample were transferred into the first well of the preloaded extraction plate before starting the extraction program.

### DENV serotyping reverse transcription-insulated isothermal polymerase chain reaction

The four DENV serotyping RT-iiPCR reagents (POCKIT Dengue Virus Serotype 1 Reagent Set, POCKIT Dengue Virus Serotype 2 Reagent Set, POCKIT Dengue Virus Serotype 3 Reagent Set, POCKIT Dengue Virus Serotype 4 Reagent Set; GeneReach) were performed as described in their user manuals. Briefly, lyophilized RT-iiPCR reagent was rehydrated with 50 μl of Premix buffer (GeneReach), and 5 μl of sample was added to the mixture. Subsequently, 50 μl of the final mixture were transferred to an R-tube (GeneReach), which was spun briefly in a cubee mini centrifuge (GeneReach), The R-tubes were placed into the reaction chamber of a POCKIT Nucleic Acid Analyzer or a hand-held POCKIT Micro Plus Nucleic Acid Analyzer, and a run was initiated. The default program, including an RT step at 50°C for 10 min and an iiPCR step at 95°C for about 30 min, was completed in less than 1 h. Results based on signal-to-noise (S/N) ratios according to the default S/N thresholds used by the built-in algorithm [35] were shown on the display screen at the end of the program.

### CDC DENV-1–4 real-time reverse transcription-polymerase chain reaction

To evaluate the performance of the four DENV serotyping RT-iiPCR reagents in detecting DENV in serum samples, side-by-side comparison with the multiplex CDC DENV-1-4 Real Time RT-PCR Assay (reference qRT-PCR) [36] was performed. The reference qRT-PCR assay includes 4 sets of oligonucleotide primers and 4 dually labeled 5’ fluorescent TaqMan probes to differentiate the four serotypes. The reaction was performed with a SuperScript III Platinum One-Step qRT-PCR kit (Invitrogen, Carlsbad, CA, USA) without 6-carboxy-X-rhodamine in a Magnetic Induction Cycler (MIC, Bio Molecular System, Upper Coomera, Queensland, Australia). Each reaction included 5 μl of the sample nucleic acid. Signals from the DENV-1, -2, -3, and -4 probes were collected using the 6-carboxyfluorescein, hexachlorofluorescein, Texas red, and Cy5 channels, respectively. The thermocycling program included an RT step at 50°C for 30 min, followed by 95°C for 2 min and 45 cycles of denaturation at 95°C for 15 s and annealing at 60°C for 1 min. Samples generating a threshold cycle (*CT*) value were considered positive.

### Statistical analysis

The degree of agreement between two assays was assessed by calculating Cohen’s kappa values.

## Results

### Analytical sensitivity of DENV-1, -2, -3, -4 serotyping RT-iiPCR

The detection endpoints of the DENV-1, -2, -3, -4 serotyping RT-iiPCR reagents were evaluated by side-by-side comparison with the reference multiplex qRT-PCR by using their respective target DENV serotypes. 10-fold serial dilutions (100, 10, 1, 0.1, and 0.01 PFU/ml) of each isolate were made in DENV-negative human serum and each was subjected to nucleic acid extraction in triplicate. The results are summarized in Table 1. The 100% detection endpoints were found at 10 and 1 PFU/ml DENV-1 with the reference qRT-PCR and DENV-1 RT-iiPCR, respectively, at 1 PFU/ml DENV-2 with both qRT-PCR and DENV-2 RT-iiPCR, at 1 PFU/ml DENV-3 with both qRT-PCR and DENV-3 RT-iiPCR, and at 10 and 1 PFU/ml DENV-4 with the qRT-PCR and DENV-4 RT-iiPCR, respectively. All data indicated that the four DENV-1, -2, -3, and -4 RT-iiPCR had analytical sensitivity comparable to that of the reference qRT-PCR in detecting their target DENV serotypes.

**Table 1:** Analytical Sensitivity of dengue virus serotype 1-, 2-, 3-, and 4-specific RT-iiPCR reagents.

RT-iiPCR	Strain	Titer (PFU/ml)	Detection rate (no. positive/no. total)	
			Reference qRT-PCR	RT-iiPCR
DENV-1	DENV-1	10^3^	3/3	3/3
		10^2^	3/3	3/3
		10^1^	3/3	3/3
		10^0^	0/3	3/3
		10^−1^	0/3	2/3
		10^−2^	0/3	0/3
DENV-2	DENV-2	10^3^	3/3	3/3
		10^2^	3/3	3/3
		10^1^	3/3	3/3
		10^0^	3/3	3/3
		10^−1^	2/3	0/3
		10^−2^	0/3	0/3
DENV-3	DENV-3	10^3^	3/3	3/3
		10^2^	3/3	3/3
		10^1^	3/3	3/3
		10^0^	3/3	3/3
		10^−1^	0/3	1/3
		10^−2^	0/3	0/3
DENV-4	DENV-4	10^3^	3/3	3/3
		10^2^	3/3	3/3
		10^1^	3/3	3/3
		10^0^	0/3	3/3
		10^−1^	0/3	1/3
		10^−2^	0/3	0/3

DENV, dengue virus; RT-iiPCR, reverse transcription-insulated isothermal polymerase chain reaction; PFU, plaque forming unit; qRT-PCR, real-time reverse transcription-polymerase chain reaction; boxed, 100% detection end point.

### Analytical specificity of DENV-1, -2, -3, -4 serotyping RT-iiPCR

To assess the specificity of each DENV serotyping RT-iiPCR reagent, the four DENV serotypes and two other viruses (Zika virus MR766, Zika virus PRVABC59, and chikungunya virus CK9500004) known to cause febrile illness or skin rash illness were tested. All four singleplex DENV serotyping RT-iiPCR reagents did not react with the other three non-targeted dengue virus serotypes, Zika virus and chikungunya virus in the exclusivity test panel (Table 2), indicating that the reagents had excellent specificity for their target DENV serotypes.

**Table 2:** Analytical specificity of dengue virus serotype 1-, 2-, 3-, and 4-specific RT-iiPCR reagents.

Pathogen	Titer (PFU/ml)	RT-iiPCR			
		DENV-1	DENV-2	DENV-3	DENV-4
DENV1 8/6	1.8 x 10^3^	positive	negative	negative	negative
DENV2 99.8.30	2.1 x 10^6^	negative	positive	negative	negative
DENV3 5/12	1.0 x 10^6^	negative	negative	positive	negative
DENV4 1021021	1.9 x 10^5^	negative	negative	negative	positive
ZIKV PRVABC59	2.5 x 10^4^	negative	negative	negative	negative
ZIKV MR766	1.2 x 10^5^	negative	negative	negative	negative
CHIKV	1.4 x 10^5^	negative	negative	negative	negative

DENV, dengue virus; ZIKV, Zika virus; CHIKV, chikungunya virus; PFU, plaque forming unit; RT-iiPCR, reverse transcription-insulated isothermal polymerase chain reaction.

### Clinical performance of DENV-1, -2, -3, -4 serotyping RT-iiPCR

To evaluate the clinical performance of each DENV serotyping RT-iiPCR reagent, 40 serum samples (about 20 DENV serotype-positive and 20 DENV-negative) were tested for each respective reagent. For this purpose, 20 DENV-1, 20 DENV-2, 20 DENV-3, and 20 DENV-negative samples previously identified by a real-time PCR [34] were used. Due to the lack of DENV-4 positive clinical samples in the region, DENV-4 samples were prepared by spiking 20 DENV-negative human serum specimens with different concentrations of the DENV-4 DN9000475A stock (1.9 x 10^5^ PFU/ml). The samples were subjected directly to nucleic acid extraction by the taco mini method. Nucleic acid extracts were tested by the respective DENV serotyping RT-iiPCR reagents and the reference multiplex qRT-PCR in parallel. Completely matched results were found for DENV-1 detection (20 positive and 20 negative) between the DENV-1 RT-iiPCR and the reference qRT-PCR, as well as for DENV-4 detection (20 positive and 20 negative) between the DENV-4 RT-iiPCR and the qRT-PCR (Table 3). 20 and 19 samples were DENV-2 positive and negative, respectively, by both the DENV-2 RT-iiPCR and the reference assays; whereas one sample was DENV-2 negative by the index assay but positive by the reference assay (Ct = 44.49, Table 3). Similarly, 20 and 19 samples were determined to be DENV-3 positive and negative, respectively, by both the DENV-3 RT-iiPCR and reference assays; one sample was DENV-3 negative by the qRT-PCR but positive by the RT-iiPCR (Table 3). Therefore, compared to the reference qRT-PCR, the DENV-1 RT-iiPCR and DENV-4 RT-iiPCR had 100% overall agreement (CI95%, 97.3–100%), the DENV-2 RT-iiPCR had 97% overall agreement (CI95%, 89.8–97.5%) and the DENV-3 RT-iiPCR had 97% overall agreement (CI95%, 89.8–97.5%), indicating that the four DENV serotyping RT-iiPCR reagents on the POCKIT system had clinical performance comparable to those of the reference qRT-PCR to detect their target DENV serotypes in serum samples.

**Table 3:** Clinical performance characteristics of dengue virus serotypes 1-, 2-, 3-, and 4-specific RT-iiPCR reagents on POCKIT nucleic acid analyzer for the detection of dengue virus in human serum.

Assay and Result	Reference qRT-PCR			% Specificity (95% CI)	% Sensitivity (95% CI)	% Agreement (95% CI)
	Positive	Negative	Total			
**DENV-1 RT-iiPCR**	DENV-1			100% (88 ~ 100%)	100% (88 ~ 100%)	100% (93.7 ~ 100%)
Positive	20	0	20			
Negative	0	20	20			
Total	20	20	40			
**DENV-2 RT-iiPCR**	DENV-2			100% (87.5 ~ 100%)	95.2% (81.5 ~ 100%)	97.5% (89.8 ~ 100%)
Positive	20	0	20			
Negative	1	19	20			
Total	21	19	40			
**DENV-3 RT-iiPCR**	DENV-3			95% (80.6 ~ 100%)	100% (88.1 ~ 100%)	97.5% (89.8 ~ 100%)
Positive	20	1	21			
Negative	0	19	19			
Total	20	20	40			
**DENV-4 RT-iiPCR**	DENV-4			100% (88 ~ 100%)	100% (88 ~ 100%)	100% (93.7 ~ 100%)
Positive	20	0	20			
Negative	0	20	20			
Total	20	20	40			

DENV, dengue virus; RT-iiPCR, reverse transcription-insulated isothermal polymerase chain reaction; qRT-PCR, real-time reverse transcription-polymerase chain reaction; CI, confidence interval.

### Serotype-specific detection of dengue virus serotypes 1–4 in mosquitos

The combination of the hand-held POCKIT Micro Plus and the compact taco mini is available in a suitcase for pathogen surveillance at points of need. With the four serotyping RT-iiPCR reagents, it will be possible to performed DENV serotyping soon after the mosquitoes are trapped on site even at settings of limited resources. Here, we evaluated preliminarily the performance of the four serotyping RT-iiPCR reagents to detect their target DENV serotypes in mosquito specimens on the hand-held POCKIT Micro Plus PCR system. Nucleic acids extracted from female *A*. *aegypti* mosquitoes experimentally infected with DENV-1, -2, -3, and -4 serotype were subjected to PCR testing by the POCKIT DENV-1, -2, -3, or -4 RT-iiPCR. The results showed that the DENV-1, -2, -3, and -4 RT-iiPCR can detect their target DENV serotypes but not the serotypes to be excluded (Table 4), indicating excellent specificity for DENV serotypes in mosquito sample matrix.

**Table 4:** Detection of dengue virus serotypes 1, 2, 3, and 4 in mosquito samples by dengue virus serotype 1-, 2-, 3-, and 4-specific RT-iiPCR reagents on POCKIT nucleic acid analyzer.

DENV-infected mosquitos (serotype)	RT-iiPCR			
	DENV-1	DENV-2	DENV-3	DENV-4
DENV-1	positive	negative	negative	negative
DENV-2	negative	positive	negative	negative
DENV-3	negative	negative	positive	negative
DENV-4	negative	negative	negative	positive

DENV, dengue virus; RT-iiPCR, reverse transcription-insulated isothermal polymerase chain reaction; qRT-PCR.

## Discussion

There is an urgent need for better surveillance and control of DENV spread to help mitigate the global spread of epidemic dengue. Accurate and rapid detection and serotyping of DENV can help improve the recognition of severe dengue warning signs in patients suspected of DENV infection. Timely reporting on the detection and serotyping status of DENV in mosquito can serve as an alert to people who fall ill to consider the possibility of dengue infection and seek medical assistant in time, and to initiate timely community and vector control programs to help mitigate the spread of DENV infection.

In this study, tests with clinical serum samples showed that the four singleplex serotyping RT-iiPCR reagents had clinical performance comparable to that of the reference qRT-PCR for the detection of their target serotypes in human serum. There was one qRT-PCR-positive/RT-iiPCR-negative sample for DENV-2 and qRT-PCR-negative/RT-iiPCR-positive for DENV-3. The discrepancy in detection between the two assays was likely due to low viral loads in these samples; one supporting observation was that the one qRT-PCR-positive/RT-iiPCR-negative sample had a Ct of 44.49 in qRT-PCR. The four index reagents offered excellent analytical sensitivity and specificity to detect their target DENV serotypes in human serum on the compact field-deployable POCKIT device, and also had great analytical specificity in mosquito samples on the hand-held POCKIT Micro Plus.

In DENV diagnosis, DENV serotyping is also important since DENV-2 and DENV-3 are more often associated with severe diseases than the other serotypes [6]. Furthermore, when patients with previous DENV infection were infected with a heterotypic serotype, the chances for them to develop DHF-DSS were elevated [7]. The pan-DENV RT-iiPCR/POCKIT system validated previously for the detection of all four DENV serotypes in human plasma and serum [32, 33] is useful in aiding the identification of acute DENV infection, especially for remote regions with high burdens of DENV infection. However, this system could not differentiate between different DENV serotypes. In this study, we showed that the four new RT-PCR reagents for DENV serotyping can work on the same field-deployable PCR system to serve as tools to allow timely near-patient serotyping of DENV in human and mosquitos to facilitate efficient disease management and public health surveillance.

As shown in Table 1, similar to that of the reference qRT-PCR for all four serotypes, the sensitivity of the RT-iiPCR system was at biological titers of around 10^0^ PFU/ml. This was consistent to the performance of other molecular detection methods for DENV [37–39]. As reported previously, RNA copy numbers were likely significantly higher than PUFs, due to defective virus particles or viral RNA freed from infected cells in the sample matrix [40–42].

To aid laboratory confirmation of DENV infection during the first 5 to 6 days after symptomatic onset, detection methods for DENV RNA have been recommended by WHO [1]. Among them, real-time RT-PCR allows serotyping of DENV. However, this technology is in general not available at most PONs to provide timely serotyping results in regions with threats of epidemic dengue; RNA degradation during the shipping process to the central laboratories is also a concern [43]. The POCKIT or POCKIT Micro Plus device has been bundled with the field-deployable taco mini extraction system in a durable suitcase (POCKIT combo, POCKIT Micro combo, respectively; GeneReach) to meet the needs of PON applications at different settings. The equipment can be operated with a car battery or a rechargeable battery and only a few simple steps are needed from sample to results with this mobile PCR system.

Current commercially available NS1 immunological test products are rapid and do not require trained personnel to operate. They have been shown to have great performance for detecting DENV in both human and mosquitoes [44]. However, they do not provide serotype information. In addition, its sensitivity was relatively low on days 1 and 2 and after day 5 post-symptomatic onset in human, compared to that seen with the-RT-PCR methods [16].

In conclusion, performed on the portable POCKIT system, the four POCKIT singleplex serotyping RT-iiPCR reagents have potential to serve as a relatively inexpensive, rapid, and simple PON tool for early detection and serotyping of DENV in viremic patients as well as in infected mosquitoes, enabling timely management and control of dengue disease in underserved communities. Studies to verify and validate further the performance of these reagents on the mobile PCR laboratory system for DENV subtyping in both human and mosquitoes are underway.
