# Correction: An RT-PCR panel for rapid serotyping of dengue virus serotypes 1 to 4 in human serum and mosquito on a field-deployable PCR system

**DOI:** 10.1371/journal.pone.0315046

**Published:** 2024-12-03

**Authors:** Jih-Jin Tsai, Wei-Liang Liu, Ping-Chang Lin, Bo-Yi Huang, Ching-Yi Tsai, Pin-Hsing Chou, Fu-Chun Lee, Chia-Fong Ping, Pei-Yu Alison Lee, Li-Teh Liu, Chun-Hong Chen

The first and eleventh authors, Jih-Jin Tsai and Chun-Hong Chen, should not have been attributed equal contribution to this work.
